# Development and validation of an endoplasmic reticulum stress long non-coding RNA signature for the prognosis and immune landscape prediction of patients with lung adenocarcinoma

**DOI:** 10.3389/fgene.2023.1024444

**Published:** 2023-02-20

**Authors:** Jie Zeng, Zhenyu Wu, Meijuan Luo, Xie Xu, Wenjie Bai, Guijing Xie, Quhai Chen, Dengfeng Liang, Zixun Xu, Mindong Chen, Jianjiang Xie

**Affiliations:** ^1^ Department of Thoracic Surgery, Guangzhou First People’s Hospital, South China University of Technology, Guangzhou, China; ^2^ Department of Urology, The First People’s Hospital of Foshan, Foshan, China; ^3^ Sun Yat-sen University Cancer Center, State Key Laboratory of Oncology in South China, Collaborative Innovation Center for Cancer Medicine, Guangzhou, China; ^4^ Department of Radiology, Sun Yat-sen University Cancer Center, Guangzhou, China

**Keywords:** lung adenocarcinoma, long non-coding RNA, endoplasmic reticulum stress, prognostic model, immune landscape

## Abstract

**Background:** Lung adenocarcinoma (LUAD), the most common histotype of lung cancer, may have variable prognosis due to molecular variations. This work investigated long non-coding RNA (lncRNA) related to endoplasmic reticulum stress (ERS) to predict the prognosis and immune landscape for LUAD patients.

**Methods:** RNA data and clinical data from 497 LUAD patients were collected in the Cancer Genome Atlas database. Pearson correlation analysis, univariate Cox regression, least absolute shrinkage and selection operator regression analyses, as well as the Kaplan-Meier method, were used to screen for ERS-related lncRNAs associated with prognosis. The risk score model was developed using multivariate Cox analysis to separate patients into high- and low-risk groups and a nomogram was constructed and evaluated. Finally, we explore the potential functions and compared the immune landscapes of two groups. Quantitative real-time PCR was used to verify the expression of these lncRNAs.

**Results:** Five ERS-related lncRNAs were shown to be strongly linked to patients’ prognosis. A risk score model was built by using these lncRNAs to categorize patients based on their median risk scores. For LUAD patients, the model was found to be an independent prognostic predictor (*p* < 0.001). The signature and clinical variables were then used to construct a nomogram. With 3-year and 5-year OS’ AUC of 0.725 and 0.740, respectively, the nomogram’s prediction performance is excellent. The 5-lncRNA signature was associated with DNA replication, epithelial-mesenchymal transition, and the pathway of cell cycle, P53 signaling. Between the two risk groups, immune responses, immune cells, and immunological checkpoints were found to be considerably different.

**Conclusion:** Overall, our findings indicate that the 5 ERS-related lncRNA signature was an excellent prognostic indicator and helped to predict the immunotherapy response for patients with LUAD.

## 1 Introduction

Lung cancer is the cancer with the highest fatality rate in the world, with around 25% of cancer patients dying of it (Sung et al., 2021). Lung adenocarcinoma (LUAD) is the most common histotype of lung cancer, accounting for around 40% of all lung malignancies with an increasing prevalence (Zappa and Mousa, 2016). Despite the employment of a variety of treatments, including surgery, chemoradiotherapy, targeted therapy, and immunotherapy, lung cancer patients had a dismal prognosis, with 5-year survival rates ranging from 4% to 17% (Hirsch et al., 2017). Due to molecular variations, patients with histologically identical malignancies may have a different prognosis. Therefore, compared to a single biomarker, integrating multiple biomarkers into a signature to predict the prognosis and high-risk group of patients with LUAD is of great significance. As a result, combining molecular biomarkers into a signature for predicting the prognosis of LUAD patients is of great importance.

Long non-coding RNAs (lncRNAs) have a role in a variety of physiological and pathological processes, including tumor progression and metastasis (Hu et al., 2018; Chi et al., 2019). LncRNAs are important in multi-gene regulatory networks, and they can be utilized to diagnose and predict survival in a variety of cancers (Van Leeuwen and Mikkers, 2010; Jiang et al., 2016). For example, upregulated lncRNA LINC00691 was a poor prognostic predictor for patients with lung cancer, and through modulation of SATB2, the downregulated lncRNA SATB2-AS1 inhibits tumor metastasis in colorectal cancer (Xu et al., 2019). Increasing researches suggested that lncRNA-based signatures can accurately predict overall survival (OS) in LUAD patients (Lin et al., 2018; Ren et al., 2021).

Endoplasmic reticulum stress (ERS) is defined as an imbalance in endoplasmic reticulum homeostasis, which includes the unfolded protein response and perturbation in calcium (Ca^2+^) (Hetz, 2012; Leprivier et al., 2015). Relevant studies have shown that ERS has a major part in the genesis and progression of a variety of human malignancies (Obacz et al., 2017; Da Silva et al., 2020). ERS have a good predictive value as prognostic indicators in diffuse gliomas and hepatocellular carcinoma, according to previous researches (Liu et al., 2021a; Huang et al., 2021). According to previous research achievements, ERS has been linked to drug-induced apoptosis in lung carcinoma (Joo et al., 2007). However, few sequence-based studies have examined the characteristics of lncRNA related with ERS and their relationship to OS in patients with LUAD.

By analyzing lncRNA expression data from the Cancer Genome Atlas (TCGA), we were able to identified ERS-related lncRNAs. Then, to appropriately assess patients’ prognosis, we developed a predictive multi-lncRNA signature of differentially expressed ERS-related lncRNAs.

## 2 Materials and methods

### 2.1 Identification of ERS-related lncRNAs

We access the TCGA database to obtain RNA-seq (Fragments Per Kilobase Million format) and clinical data for 497 patients with LUAD (https://cancergenome.nih.gov/). RNA-seq data was normalized by limma R package, and the mean value was used if the genes were duplicated. Two hundred and fifty-two ERS-related genes were retrieved by investigating previous studies (Supplementary Table S1) (Huang et al., 2021).

To identify the differentially expressed lncRNAs as well as the genes related to ERS between LUAD and adjacent normal tissues, we then used the criterion | log2FC | > 0.5 and False Discovery Rate (FDR) < 0.05. Pearson correlation analysis was utilized to screened out ERS-related lncRNAs, and then we employed univariate Cox regression to explore lncRNAs related prognosis. In order to narrow the range of prognosis-related lncRNAs, the least absolute shrinkage and selection operator (LASSO) regression and Kaplan-Meier analysis were used.

### 2.2 Establishment of the risk score model

Following that, to establish the optimal risk model with ERS-related lncRNAs, we conducted a multivariate Cox regression analysis and made use of its regression coefficient. The risk score was calculated using linear integration of the regression coefficient and the expression value of 5 ERS-related lncRNAs. As a result, we computed the risk score as follow:

Risk score = $\left.\overset{5}{\underset{i=1}{\sum }}\left(\beta_{i}\right.\times Exp_{i}\right)$ .

The coefficient (*βi*) was the corresponding lncRNAs’ regression coefficient of the above Cox regression model. The expression level of the identified ERS-related lncRNAs is referred to as *Expi*. This formula was used for each patient to calculate his corresponding risk score. Furthermore, we considered median risk score as the cutoff value to divide all the patients into high- and low-risk groups. The Kaplan-Meier survival curve and log-rank test were applied to demonstrate the prognostic difference between the two groups.

### 2.3 Construction and evaluation of the nomogram

Using both univariate and multivariate Cox regression analysis, the prognostic relationship between the risk score and clinical features (including age, gender, and TMN stage) was investigated. Following that, in order to predict the prognosis of each patient with LUAD more accurately, we created a nomogram for 3- and 5-year OS by using the results of multivariate Cox regression model. To evaluate the prediction performance of the nomogram, we then employed the receiver operating characteristic (ROC) curve analysis and the calibration curves.

### 2.4 Gene set enrichment and immunity landscape analysis

Gene set enrichment analysis (GSEA) was performed for the low- and high-risk groups to investigate the potential pathways and functions of these ERS-related lncRNAs. Not only Gene Ontology (GO) enrichment analysis but also Kyoto Encyclopedia of Genes and Genomes (KEGG) pathway analysis were conducted. Furthermore, we compared the differences immune cell abundance, immune function, and immune checkpoints between high- and low risk groups.

### 2.5 RNA extraction and quantitative real-time PCR

Human LUAD cells (A549, PC-9 and SKLU1) and normal bronchial epithelial cells (16HBE) were obtained from Procell Life Science and Technology (Wuhan, China). Using TRIzol reagent (BioTeke, Beijing, China), we isolated the whole RNA of the LUAD cells. Subsequently, qRT-PCR was performed utilizing the HiScript II Q RT SuperMix for qPCR (Bioer Technology, Hangzhou, China), according the instructions provided by the manufacture for all program steps. The 2^−△△Ct^ method was used to calculate the relative expression of each lnRNA. We utilized the unpaired *t*-test to compare the LUAD cells and normal cells in the expression level differences. Supplementary Table S2 shows the primer sequences.

### 2.6 Statistical analysis

We conducted all statistical analyses in this research using R software (Institute for Statistics and Mathematics, Vienna, Austria; https://www.r-project.org, version 4.0.2) and GraphPad Prism 8.0 software. The unpaired Wilcoxon Rank Sum test was used to assess the differences between continuous variables. The chi-square test was used to examine the relationship between categorical factors. The statistically significant setting for each analysis was two-tailed *p* < 0.05.

## 3 Results

### 3.1 Identification of ERS-related lncRNAs

**FIGURE 1:**
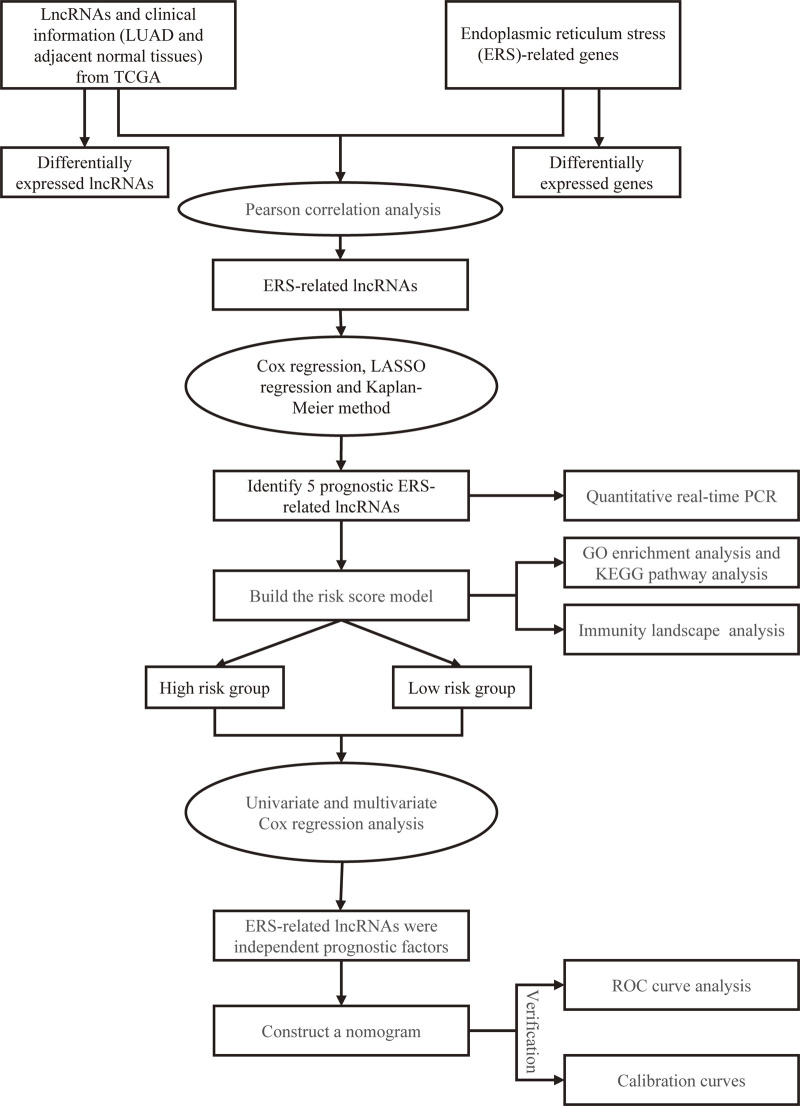
Flowchart of our design for the study.

**FIGURE 2:**
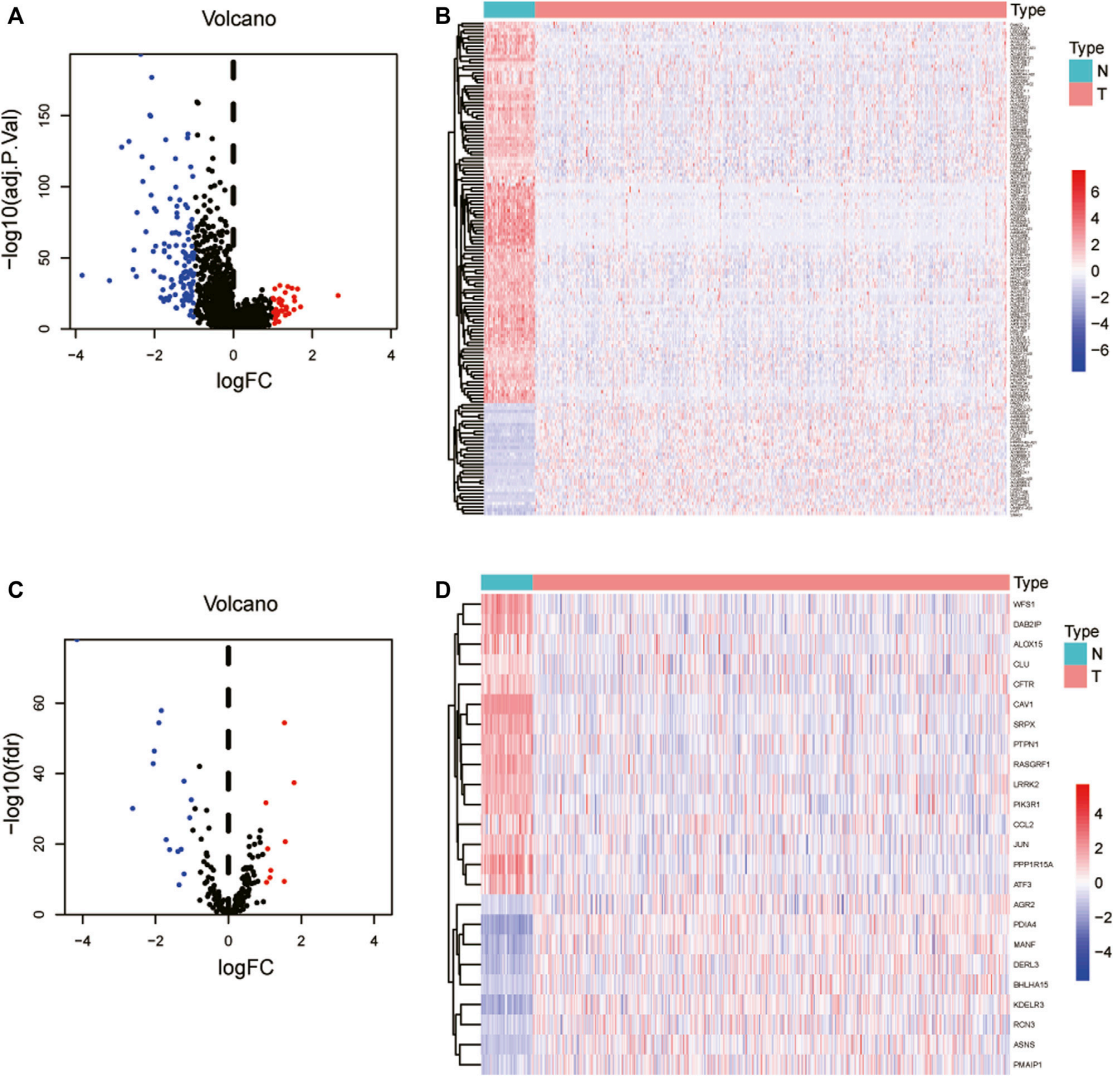
Identification of ERS-related lncRNAs in LUAD. **(A)** As shown in volcano plot, the 34 lncRNAs were upregulated in tumors, while 116 were downregulated. **(B)** Differential expression lncRNA heatmaps in tumors and normal adjacent tissues. **(C)** Nine ERS-related genes were found to be upregulated in tumors, whereas 15 were found to be downregulated. **(D)** Heatmaps of differential expression ERS-related genes in tumors and normal adjacent tissues. LUAD: Lung adenocarcinoma; ERS: Endoplasmic reticulum stress.

Figure 1 depicts the flowchart for this research. We examined 497 lung cancer patients using the TCGA database to find differentially expressed lncRNAs and their associated activities of ERS-related genes in LUAD. Subsequently, 497 tumors and 54 normal adjacent tissues were retrieved from these patients. We retrieved previous studies to extract 252 ERS-related genes in total (Huang et al., 2021). We identified that a total of 150 lncRNAs were deferentially expressed in tumors and normal adjacent tissues, with 34 being upregulated and 116 being downregulated in tumors (Figures 2A,B). A total of 24 genes were evaluated for differential expression among 252 ERS-related genes acquired from previous publications, including 9 upregulated and 15 downregulated in tumors (Figures 2C, D).

**FIGURE 3:**
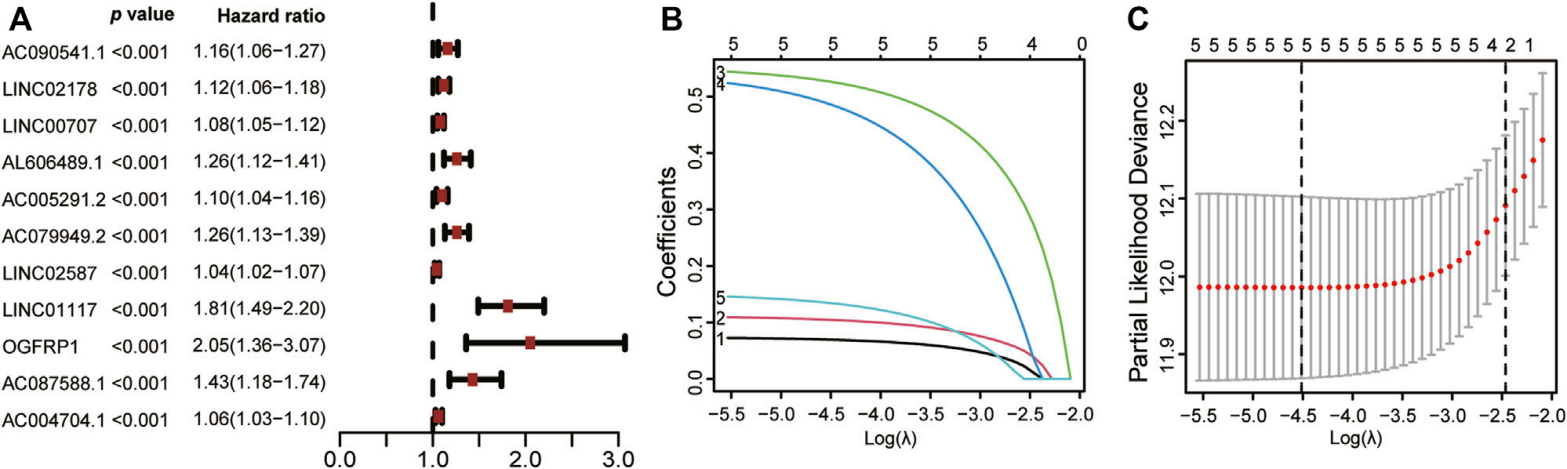
Screening ERS-related lncRNAs related to prognosis. **(A)** Based on univariate Cox analysis, 11 ERS-related lncRNAs were significantly correlated with LUAD patients’ OS, as shown in the forest plot. **(B)** LASSO analysis was utilized to filter out ERS-related lncRNAs in LUAD patients. **(C)** The LASSO coefficient spectrum for lncRNAs linked to ERS is shown. OS: Overall survival; LUAD: Lung adenocarcinoma; ERS: Endoplasmic reticulum stress; LASSO: Least absolute shrinkage and selection operator.

Furthermore, we were successful in screening out 2643 ERS-related lncRNAs utilizing Pearson correlation analysis (Supplementary Table S4). Following that, univariate Cox regression analysis showed that 11 lncRNAs were tightly associated with prognosis of LUAD patients (Figure 3A). Finally, the LASSO regression analysis and the Kaplan-Meier method incorporated 5 lncRNAs: AL606489.1, LINC02178, LINC01117, OGFRP1, and AC087588.1 (Figures 3B,C), which were found to be strongly associated to OS in lung adenocarcinoma patients (Supplementary Figure S1).

### 3.2 Establishment of the risk score model

**FIGURE 4:**
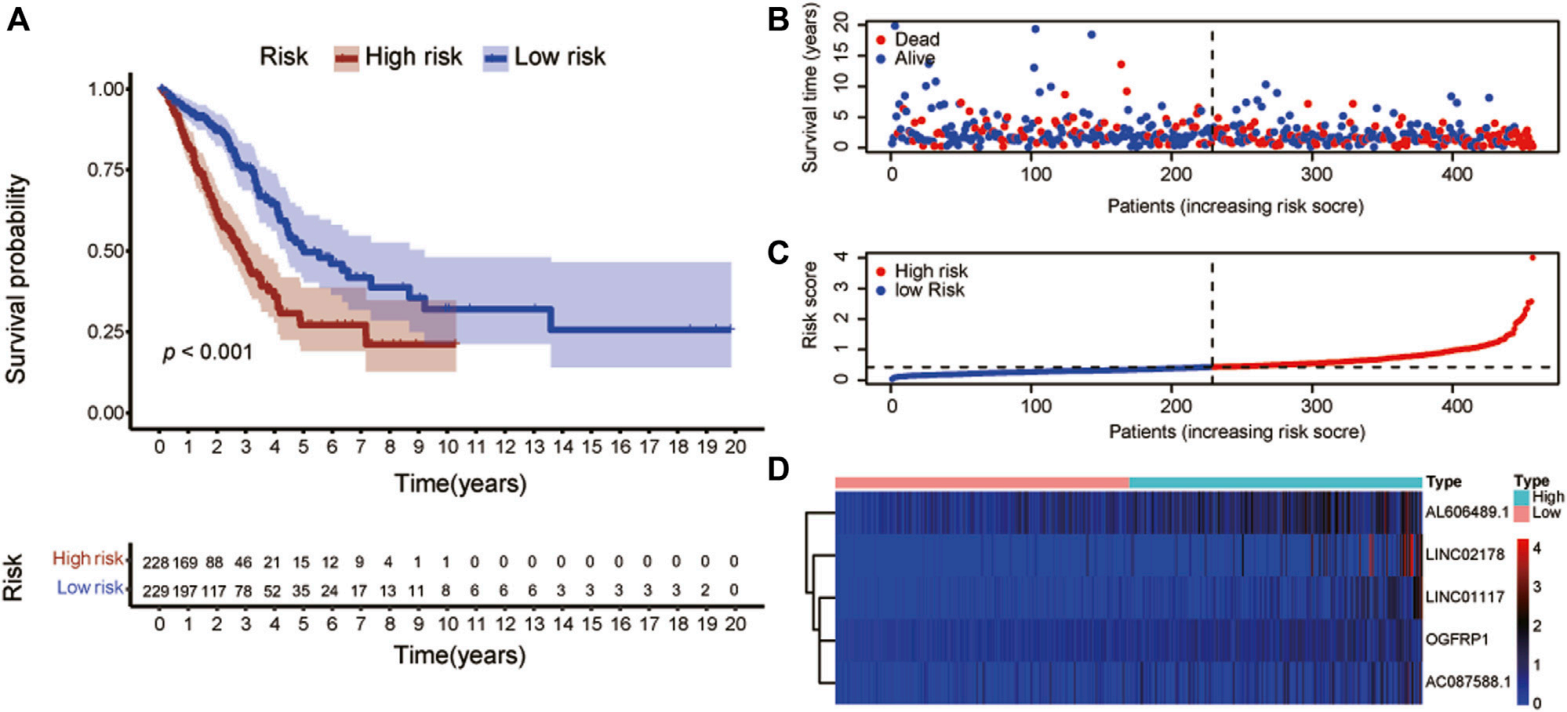
Establishment of the risk score model utilizing ERS-related lncRNAs. **(A)** Kaplan-Meier analysis showed that the patients in the high-risk group had a significantly lower overall survival than that in the low-risk group. **(B)** The survival status for each patient. **(C)** Distributions of patient risk ratings. **(D)** Heatmaps of lncRNAs associated to ERS in low- and high-risk groups. Warm colors denoted a high level of expression, whereas cold colors denoted a low level of emotion. ERS: Endoplasmic reticulum stress.

To construct risk score model, these well-characterized lncRNAs were filtered applying multivariate Cox regression. The formula was “Risk score = (0.1048) × AL606489.1 expression + (0.0697) × LINC02178 expression + (0.5262) × LINC01117 expression + (0.4863) × OGFRP1 expression + (0.1346) × AC087588.1 expression”. Based on the median risk score, the patients were separated into two parts: high-risk and low-risk. Compared with the patients in low-risk group, the patients in the high-risk group presented significantly shorter OS (*p* < 0.001, Figure 4A). The risk score distribution and survival status of these 5 lncRNAs were showed in Figures 4B,C, and the risk coefficient and patient mortality were greater in the high-risk group. The expression patterns of the 5-lncRNA signature in the two groups were revealed in the heatmap (Figure 4D).

### 3.3 Construction and evaluation of the nomogram

**FIGURE 5:**
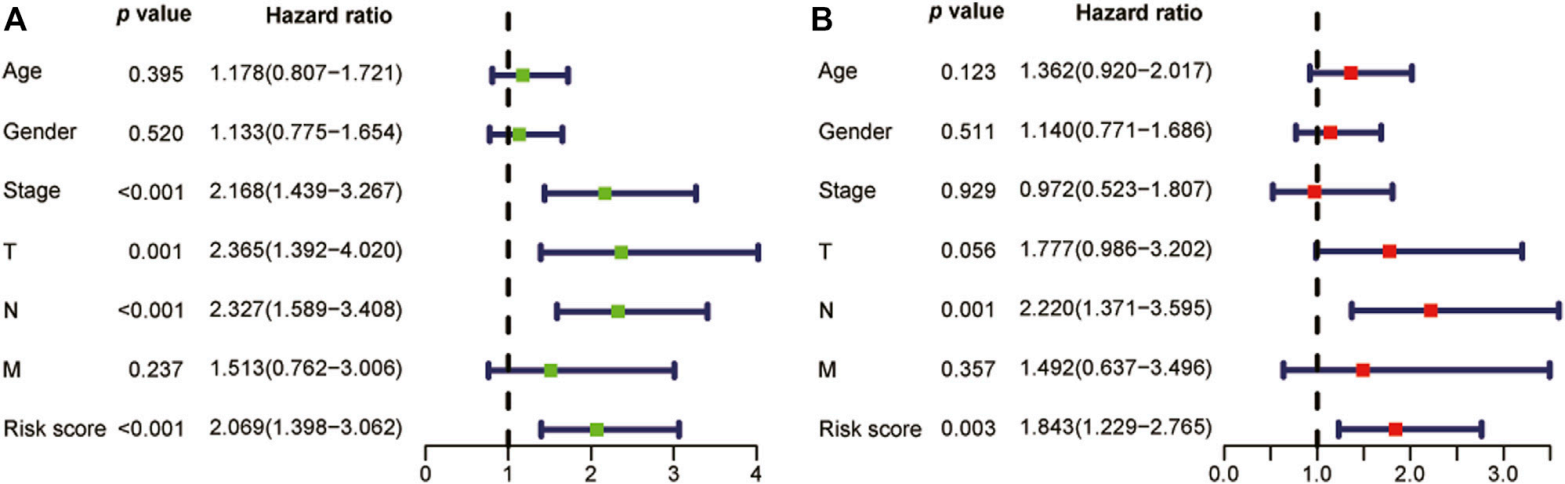
The univariate and multivariate Cox analysis analyses utilizing the risk score and clinical variables. **(A)** The univariate Cox analysis results showed that the risk score was highly associated with overall survival (*n* = 453). **(B)** In LUAD patients, the lncRNA signature, as shown in the multivariate Cox analysis, was determined to be an independent prognostic factor (*n* = 453). LUAD: Lung adenocarcinoma.

**FIGURE 6:**
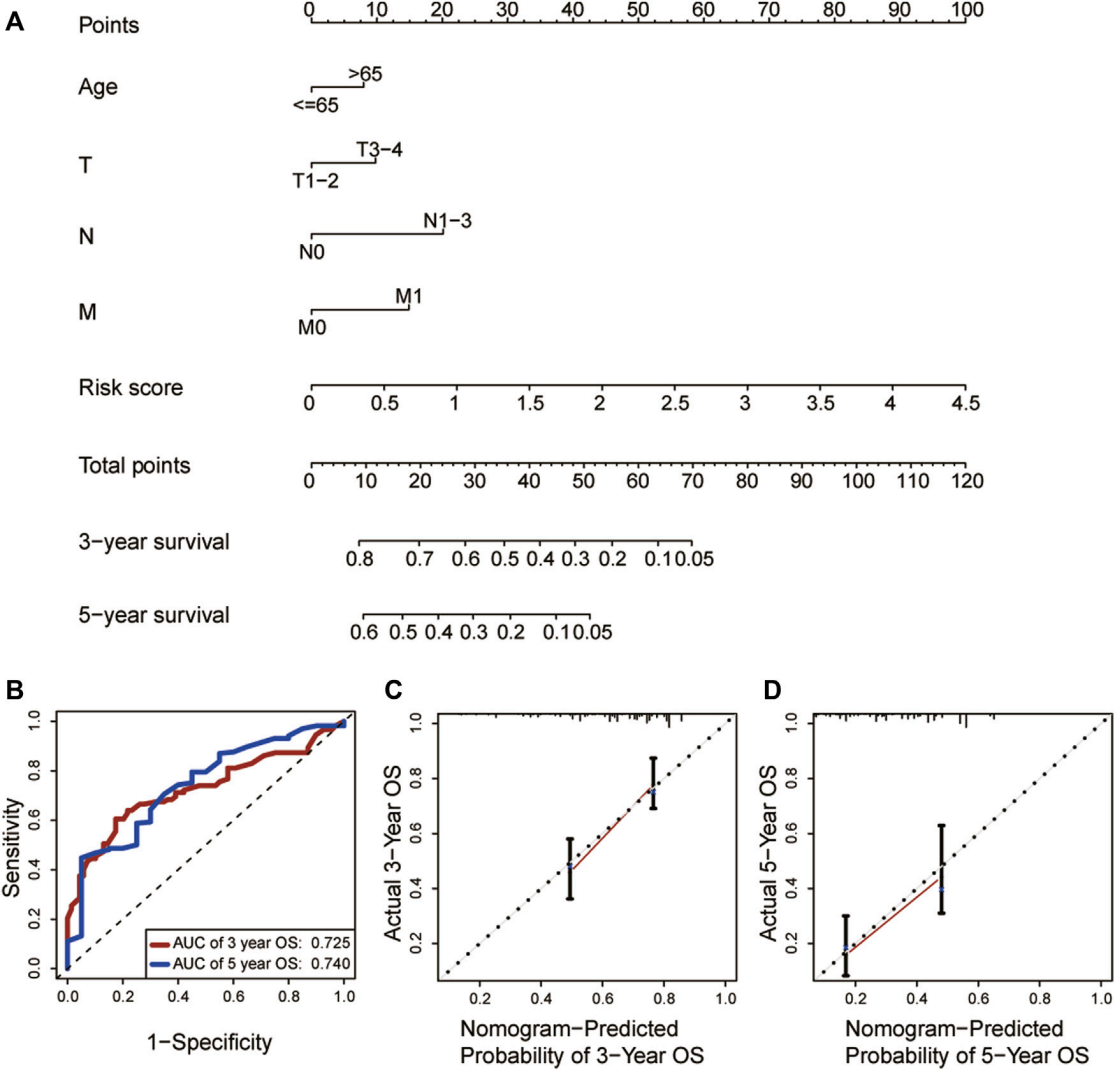
Construction and assessment of the nomogram (*n* = 453). **(A)** Establishment of the nomogram. **(B)**According to ROC curve analysis for predicting OS by the nomogram, the AUCs for 3-year OS was 0.725 and that for 3-year OS 0.740. **(C, D)** The calibration curve for nomogram’s 3-year and 5-year OS, respectively. ROC: Receiver operating characteristic; OS: Overall survival; AUC: Area under curve.

As shown in the univariate and multivariate Cox regression, the risk score was strongly related to OS (Figure 5A) and was also demonstrated to be an independent prognostic predictor of OS (Hazard ratio: 1.843; 95% Confidence interval:1.229–2.765; *p* = 0.003, Figure 5B). Next, we constructed a nomogram utilizing the results of the multivariate Cox regression (Figure 6A). The nomogram’s AUC of 3-year OS was 0.725 and that of 5-year OS was 0.740 (Figure 6B). The calibration curve also demonstrated that the nomogram with the risk score was effective in the prediction of the prognosis for patients with LUAD (Figures 6C, D).

### 3.4 Gene set enrichment analysis

**FIGURE 7:**
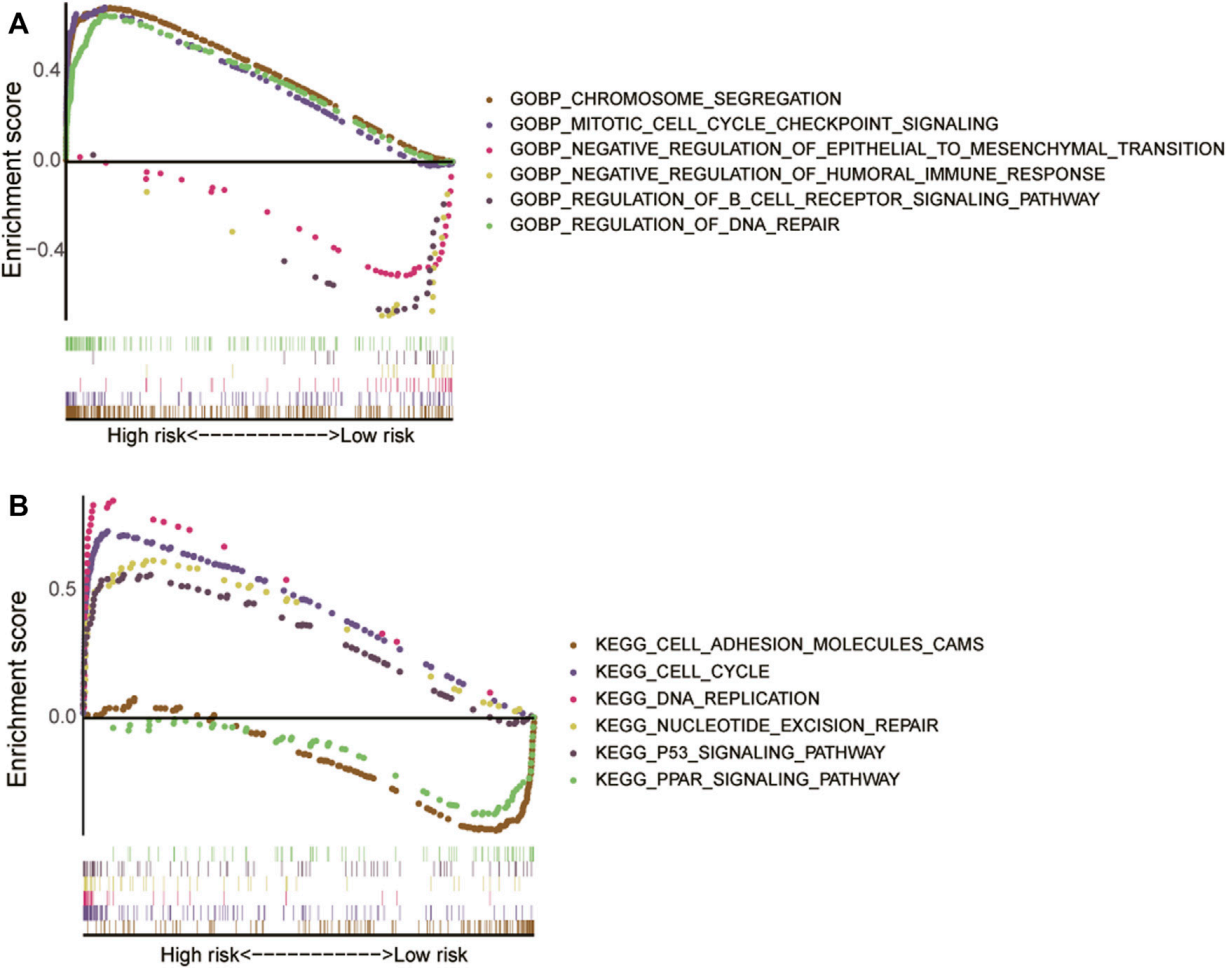
The results of Gene Set Enrichment Analysis for high- and low-risk groups. **(A)** Gene Ontology enrichment analysis. **(B)** Kyoto Encyclopedia of Genes and Genomes pathway analysis.

We found that the ERS-related lncRNAs were abundant in chromosomal segregation, mitotic cell cycle checkpoint signaling, humoral immune response, epithelial-mesenchymal transition (EMT), B cell receptor signaling pathway, and DNA repair, according to GO enrichment analysis (Figure 7A). The pathway of cell cycle, DNA replication, cell adhesion, PPAR signaling, nucleotide excision repair, and P53 signaling were shown to be related to these lncRNAs (Figure 7B). These factors may inspire people to further investigate the role of ERS-related lncRNAs in the pathogenesis further.

### 3.5 Immunity expression based on the risk model

**FIGURE 8:**
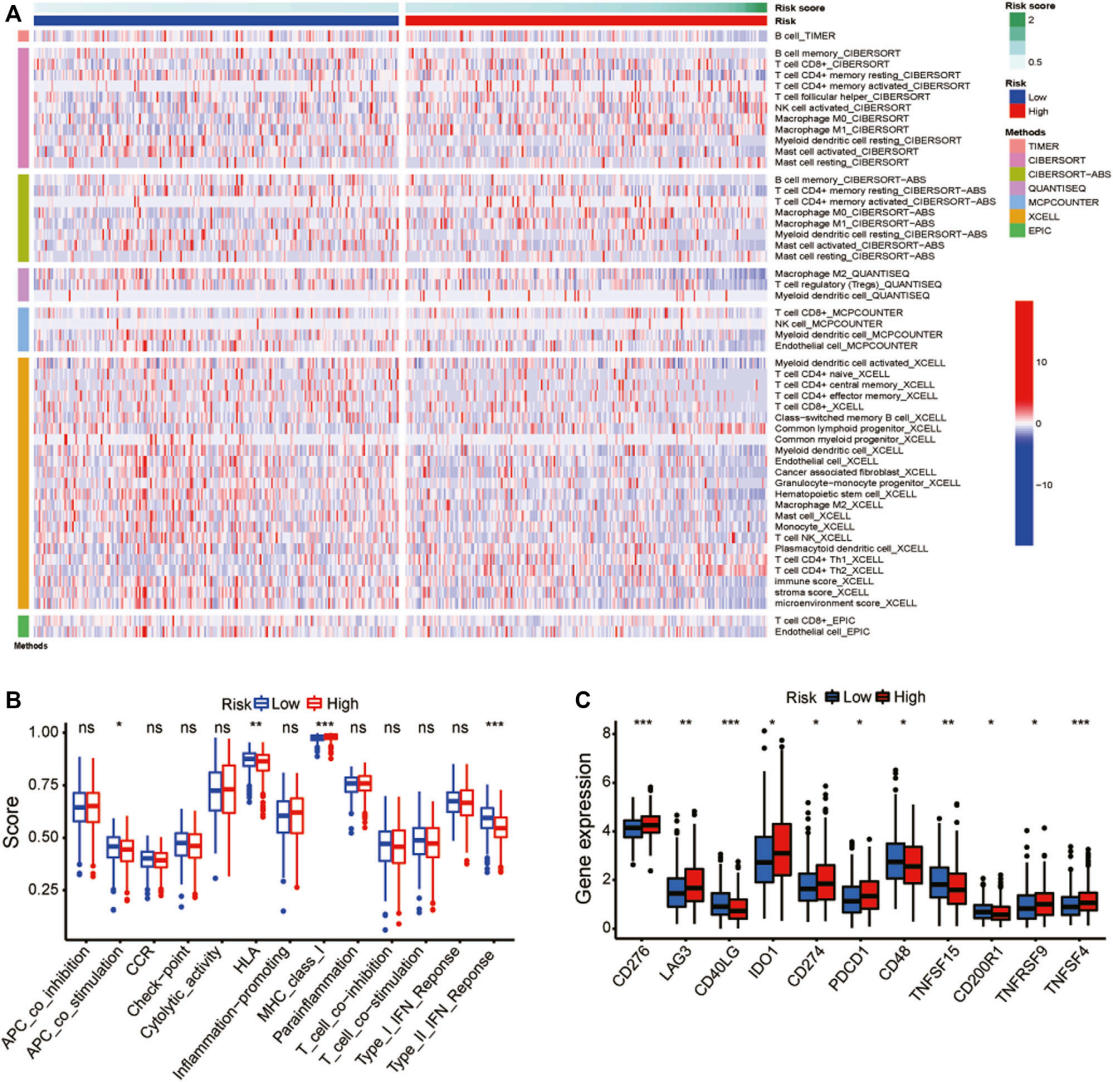
Gene expression and immune function. **(A)** A heat map for immunological responses between high- and low-risk groups. **(B)** Immune cell subpopulations and associated functions. **(C)** Expression of immune checkpoints in high-risk and low-risk groups. *p < 0.05, **p < 0.01, and ***p < 0.001; ns: no significance.

Figure 8A showed a heatmap of immune cell infiltration using several different algorithms. The immune functions were shown to be significantly different between the two groups (Figure 8B). Correlation analysis between immune cell subpopulations and related functions revealed that co-stimulation antigen-presenting cells, chemokine receptors, type II IFN response, and human leukocyte antigen showed higher expression in high-risk group than that in low-risk group, but the MHC class I was the opposite. Our research was expanded to explore the expression levels of immune checkpoint in two groups (Figure 8C). The expression of most immune checkpoints, such as CD276, LAG3 and CD48, have a lot of variation between the two groups. The results suggested that risk scores may be useful for patients with LUAD in predicting the response to immune checkpoint inhibitors (ICIs).

### 3.6 Expression level of selected lncRNAs in lung adenocarcinoma cells

**FIGURE 9:**
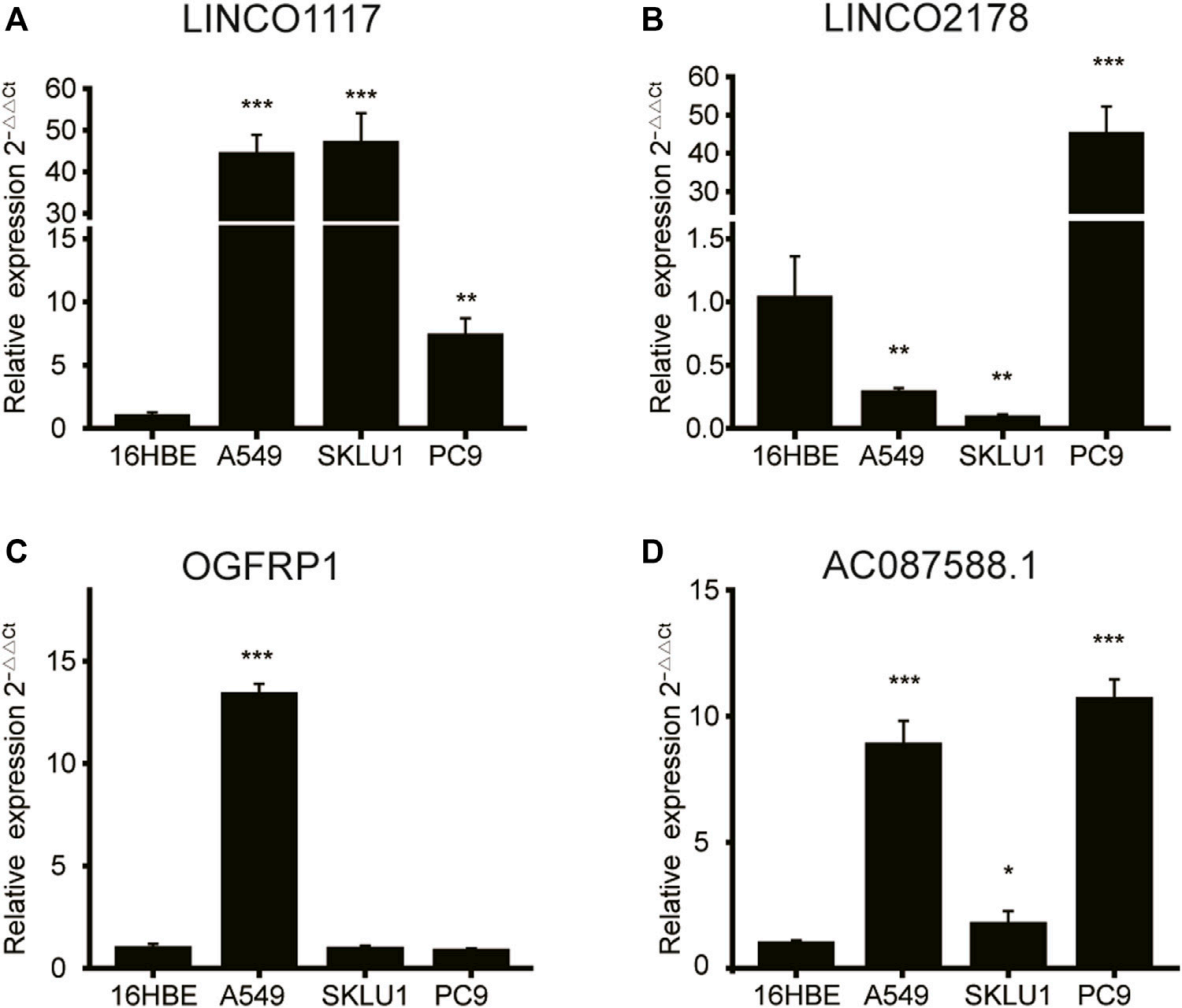
The qRT-PCR results of 4 lncRNAs’ expression in lung adenocarcinoma and normal bronchial epithelial cell lines. **(A)** LINC01117, **(B)** LINC02178, **(C)** OGFRP1, **(D)** AC087588.1. lncRNA: Long non-coding RNA. *p < 0.05, **p < 0.01, and ***p < 0.001.

Furthermore, we confirm the expression levels of 4 lncRNAs using a qRT-PCR test in the LUAD cells (A549, PC-9 and SKLU1). The expression of the lncRNA LINC01117, as shown by the results, was significantly upregulated in all the LUAD cell lines (Figure 9A). The expression of LINC02178 was significantly elevated in the PC9 cell line, but was downregulated in other cell lines (Figure 9B). OGFRP1 were obviously upregulated in the A549 cell line (Figure 9C). In all LUAD cell lines, AC087588.1 was considerably upregulated as well (Figure 9D).

## 4 Discussion

The objective of this study was to find out a better predictive marker related to ERS in order to provide approaches to develop innovative treatments for LUAD. As a result, we developed a new lncRNA signature based on ERS for the prediction of the prognosis of LUAD patients. Patients with LUAD were divided into high-risk and low-risk groups based on the signature. Patients in the low-risk group had significantly favorable OS, according to the results of the prognostic analysis. In addition, a nomogram was constructed and shown to have an excellent predictive effect. To our knowledge, this is the first study to identify and analyze prognostic lncRNAs related to ERS in patients with LUAD.

In our study, all five lncRNAs, AL606489.1, LINC02178, LINC01117, OGFRP1 and AC087588.1, were shown to be independent unfavorable prognostic indicators for LUAD. Previous research has linked AL606489.1 to ferroptosis (Guo et al., 2021), pyroptosis (Song et al., 2021) and autophagy (Wu et al., 2021), as well as a poor prognosis in LUAD patients, which is consistent with our findings. In LUAD, ERS may be associated to ferroptosis, pyroptosis, and autophagy. LINC02178 was also identified a risk factor for bladder urothelial cancer (Sun et al., 2020). Ding and Liu., 2019 discovered that OGFRP1 was a risky lncRNA in LUAD, and that OGFRP1 increased the proliferation, migration, and invasion of non-small cell lung cancer through the miR-4640–5p/eIF5A axis (Liu et al., 2021b). Furthermore, OGFRP1 acts as an oncogene in prostate cancer (Wang et al., 2021), endometrial cancer (Lv et al., 2019) and gastric cancer (Zhang et al., 2021a). However, further work is needed to investigate the biological and molecular mechanisms of these lncRNAs.

According to GSEA results, chromosome segregation, mitotic cell cycle checkpoint signaling, DNA replication, and the pathway of cell cycle, P53 signaling were abundant in the high-risk group, whereas EMT, DNA repair, and the pathway of cell adhesion, PPAR signaling were enriched in the low-risk group. Wang et al. (2016) discovered that Rac3 in lung cancer might enhance cell proliferation *via* the cell cycle pathway. A previous study found that the lncRNA PIK3CD-AS2 promotes progression in patients with LUAD by suppressing p53 signaling (Zheng et al., 2020), which implies that ERS-related lncRNA may have a similar effect. Some lncRNAs in LUAD, such as LINC0047 (Deng et al., 2020), LINC01006 (Zhang et al., 2021b) and SGMS1-AS1 (Liu et al., 2021c), have been shown to influence EMT processes.

Immune responses, immune cells, and immunological checkpoints were shown to be significantly different between the two risk groups in this study, suggesting that ERS-related lncRNAs are likely to affect the immune system in patients with LUAD. Low-risk patients with LUAD may have a stronger immunological response. ICIs, as we all know, have greatly improved the prognosis of lung cancer patients in recent years (Aldarouish and Wang, 2016). Accordingly, the efficacy of ICIs is thought to be highly linked to the immune systems of the hosts and the tumor immunological microenvironment (TIME) (Taube et al., 2018). TIME might be modulated by ERS, affecting tumor growth and response to immunotherapy (Chen and Cubillos-Ruiz, 2021). The ERS-related five-lncRNA signature in LUAD may reflect the effect of ICIs and immune response rate. Immunotherapy may be more advantageous for people with LUAD who are in the low-risk group.

There are a few shortcomings in our current study. To begin with, we were unable to gather information on more specific clinic pathological parameters, such as treatment information and tumor markers. In the future, external validation of the signature in other datasets and prospective large-scale multicenter cohorts will be required for reliability and precision. Second, due to lack of funds, the expression levels of lncRNA AL606489.1 were missing from our qPCR data. Finally, more functional experiments are needed to understand the specific mechanisms by which these ERS-related lncRNAs interact with LUAD.

## 5 Conclusion

In conclusion, the 5 ERS-related lncRNAs and a well-validated nomogram based on those lncRNAs were found to be beneficial in predicting the prognosis of LUAD patients. Our findings also point to promising avenues for LUAD immunotherapy and provide a significant foundation for future research.

## Data Availability

The datasets presented in this study can be found in online repositories. The names of the repository/repositories and accession number(s) can be found below: https://portal.gdc.cancer.gov, The Cancer Genome Atlas.

## Abbreviations

LUAD, Lung adenocarcinoma; lncRNA, Long non-coding RNA; OS, Overall survival; ERS, Endoplasmic reticulum stress; TCGA, the Cancer Genome Atlas; LASSO, Least absolute shrinkage and selection operator; ROC, Receiver operating characteristic; GSEA, Gene set enrichment analysis; GO, Gene Ontology; KEGG, Kyoto Encyclopedia of Genes and Genomes; qRT-PCR, Quantitative real-time PCR; EMT, Epithelial-mesenchymal transition; ICIs, Immune checkpoint inhibitors; TIME, Tumor immunological microenvironment.
