# Supplementary material for: Development and validation of an endoplasmic reticulum stress long non-coding RNA signature for the prognosis and immune landscape prediction of patients with lung adenocarcinoma
Source: Front Genet. 2023 Feb 20;14:1024444. doi: 10.3389/fgene.2023.1024444 (PMC9986451; doi:10.3389/fgene.2023.1024444)
Supplement: Supplementary file 1 [file Table1.DOCX]

**Table S1.** A list of endoplasmic reticulum stress-related genes.

| CAV1 |
| --- |
| WFS1 |
| PDIA4 |
| PPP1R15A |
| SRPX |
| RASGRF1 |
| ADD1 |
| DAB2IP |
| KDELR3 |
| PTPN1 |
| MANF |
| LRRK2 |
| PPP2CB |
| DNAJC18 |
| PIK3R1 |
| RNF185 |
| TLN1 |
| HDGF |
| GORASP2 |
| PDIA6 |
| ATP6V0D1 |
| ATF3 |
| DERL3 |
| GFPT1 |
| SRPRB |
| HM13 |
| ASNS |
| JUN |
| ALOX15 |
| CFTR |
| SERP2 |
| CREB3L4 |
| NFE2L2 |
| DNAJB11 |
| DCTN1 |
| DDX11 |
| HYOU1 |
| ANKZF1 |
| TRIM25 |
| PPP2R5B |
| P4HB |
| SERINC3 |
| DNAJC10 |
| NPLOC4 |
| AIFM1 |
| PREB |
| CUL7 |
| TBL2 |
| CALR |
| DNAJB12 |
| BHLHA15 |
| TRAF2 |
| ITPR1 |
| CCL2 |
| HSP90B1 |
| UGGT1 |
| SCAMP5 |
| HSPA5 |
| USP25 |
| RCN3 |
| SHC1 |
| PDIA3 |
| GET4 |
| TMBIM6 |
| AUP1 |
| BAK1 |
| ARFGAP1 |
| ATP2A2 |
| TRIB3 |
| AGR2 |
| TSPYL2 |
| TMCO1 |
| PMAIP1 |
| SDF2L1 |
| EDEM1 |
| CDK5RAP3 |
| SYVN1 |
| FKBP14 |
| CLU |
| GSK3B |
| AMFR |
| UGGT2 |
| TPP1 |
| PSMC6 |
| CTH |
| BCL2L11 |
| DNAJB14 |
| UBXN6 |
| CTDSP2 |
| YIF1A |
| NRBF2 |
| FOXRED2 |
| SSR1 |
| POMT2 |
| TMEM117 |
| UBXN4 |
| ATF4 |
| CHAC1 |
| LMNA |
| UFM1 |
| SGTB |
| UBE2G2 |
| MBTPS2 |
| ERP44 |
| RNF175 |
| RNF183 |
| GOSR2 |
| USP14 |
| BOK |
| USP19 |
| RNF186 |
| YOD1 |
| EP300 |
| EEF2 |
| BBC3 |
| TMEM33 |
| CREB3L2 |
| USP13 |
| UBAC2 |
| EIF2B5 |
| ATP2A1 |
| EXTL2 |
| RNF121 |
| EDEM3 |
| TTC23L |
| THBS1 |
| PARK7 |
| SDF2 |
| COPS5 |
| VAPB |
| MAN1B1 |
| MAGEA3 |
| TMUB1 |
| OPA1 |
| SEC31A |
| UFC1 |
| NFE2L1 |
| ATXN3 |
| ERN2 |
| MBTPS1 |
| UBQLN1 |
| DNAJB2 |
| NCK2 |
| CEBPB |
| TRIM13 |
| FAF2 |
| ERP27 |
| SIRT1 |
| PDX1 |
| UBE2K |
| NCK1 |
| ATF6B |
| TXNDC12 |
| ANKS4B |
| DERL2 |
| PDIA2 |
| TMEM129 |
| DDRGK1 |
| PPP1R15B |
| UBE2J2 |
| MAP3K5 |
| SERP1 |
| SEL1L |
| CCND1 |
| CCDC47 |
| SEC61B |
| UBQLN2 |
| UBXN8 |
| STUB1 |
| SGTA |
| BCL2 |
| WIPI1 |
| TOR1A |
| GRINA |
| FBXO6 |
| FBXO2 |
| UBE4B |
| DNAJB9 |
| BCL2L1 |
| TMTC3 |
| JKAMP |
| BRSK2 |
| PARP16 |
| STT3B |
| DDIT3 |
| THBS4 |
| EIF2AK3 |
| PLA2G6 |
| TMEM67 |
| TATDN2 |
| PML |
| ERLEC1 |
| VCP |
| EDEM2 |
| UBA5 |
| CREB3L1 |
| TP53 |
| UBE4A |
| HSPA1A |
| UBXN1 |
| IGFBP1 |
| STC2 |
| RNF5 |
| FCGR2B |
| UBE2J1 |
| ERLIN2 |
| ATF6 |
| BFAR |
| PTPN2 |
| TNFRSF10B |
| PIK3R2 |
| ZBTB17 |
| ERN1 |
| RNFT1 |
| FGF21 |
| TMUB2 |
| EIF2AK2 |
| SEC16A |
| HERPUD1 |
| RNF103 |
| TMX1 |
| APAF1 |
| CASP4 |
| SULT1A3 |
| RHBDD1 |
| PDIA5 |
| EXTL3 |
| KLHDC3 |
| RASGRF2 |
| ATG10 |
| EIF2AK4 |
| HSPA13 |
| EXTL1 |
| SESN2 |
| CREB3L3 |
| SVIP |
| ERP29 |
| OS9 |
| CXXC1 |
| DERL1 |
| ACADVL |
| FLOT1 |
| BAX |
| TARDBP |
| ERLIN1 |
| FICD |
| GSK3A |
| EIF2S1 |
| DNAJC3 |
| RNF139 |
| CREB3 |
| BCAP31 |
