# Supplementary material for: Development and validation of an endoplasmic reticulum stress long non-coding RNA signature for the prognosis and immune landscape prediction of patients with lung adenocarcinoma
Source: Front Genet. 2023 Feb 20;14:1024444. doi: 10.3389/fgene.2023.1024444 (PMC9986451; doi:10.3389/fgene.2023.1024444)
Supplement: Supplementary file 2 [file Table2.DOCX]

| Primers | Forward sequence | Reverse sequence |
| --- | --- | --- |
| β-actin | TGGCACCCAGCACAATGAA | CTAAGTCATAGTCCGCCTAGAAGCA |
| LINC01117 | ACTTTAGTGTCGAAAGCTCC | TCACTAAAACATTTGGGTAGGGG |
| LINC02178 | CCTATGAGTGAGGGTGAT | CCATGCCTACAACTCTCGTGC |
| OGFRP1 | CTCACTTGCCAGCTCTCGT | GAGTTTCCCGCTCTGTTAG |
| AC087588.1 | TCCTGCCTTCAAAATCCTCTTCG | GGGTGGGTGCATTCTGTGTG |

Table S2. Sequences of primers used in the qRT-PCR.

qRT-PCR：quantitative real-time PCR.
