# Supplementary material for: Development and validation of an endoplasmic reticulum stress long non-coding RNA signature for the prognosis and immune landscape prediction of patients with lung adenocarcinoma
Source: Front Genet. 2023 Feb 20;14:1024444. doi: 10.3389/fgene.2023.1024444 (PMC9986451; doi:10.3389/fgene.2023.1024444)
Supplement: Supplementary file 4 [file Table3.DOCX]

**Table S3. The clinical characteristics of LUAD patients in the TCGA dataset**

|  | All patients (n = 453) |
| --- | --- |
| Age (n %) |  |
| ≤65 years | 221 (48.8%) |
| >65 years | 232 (51.2%) |
| Gender (n %) |  |
| Female | 248 (54.7%) |
| Male | 205 (45.3%) |
| Overall stage (n %) |  |
| I and II | 357 (78.8%) |
| III and IV | 96 (21.2%) |
| T stage (n %) |  |
| T1 and T2 | 396 (87.4%) |
| T3 and T4 | 57 (12.6%) |
| N stage (n %) |  |
| N0 | 301 (66.4%) |
| N1, N2 and N3 | 152 (33.6%) |
| M stage (n %) |  |
| M0 | 431 (95.1%) |
| M1 | 22 (4.86%) |
