# Supplementary figures and images for: Development and validation of an endoplasmic reticulum stress long non-coding RNA signature for the prognosis and immune landscape prediction of patients with lung adenocarcinoma
Source: Front Genet. 2023 Feb 20;14:1024444. doi: 10.3389/fgene.2023.1024444 (PMC9986451; doi:10.3389/fgene.2023.1024444)

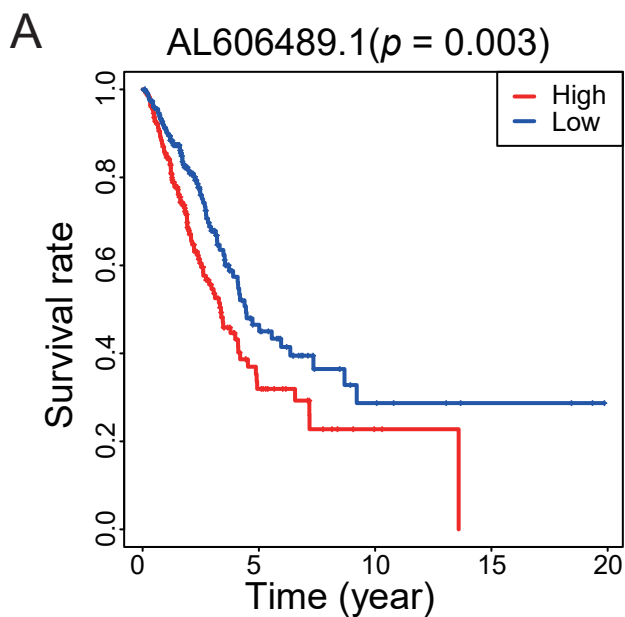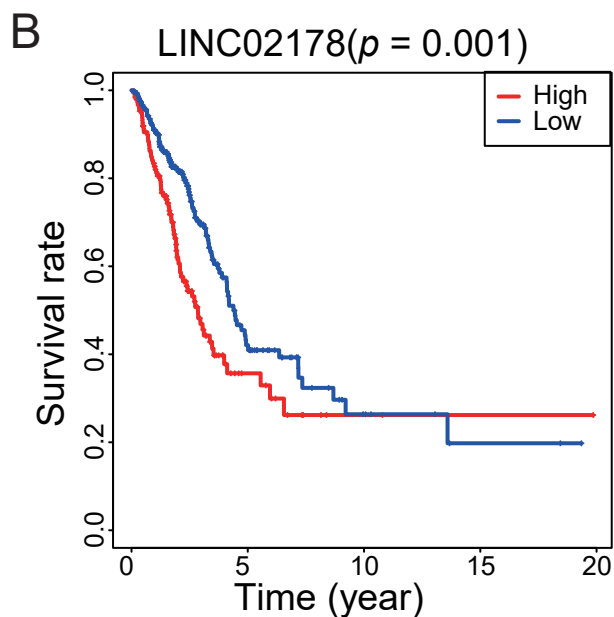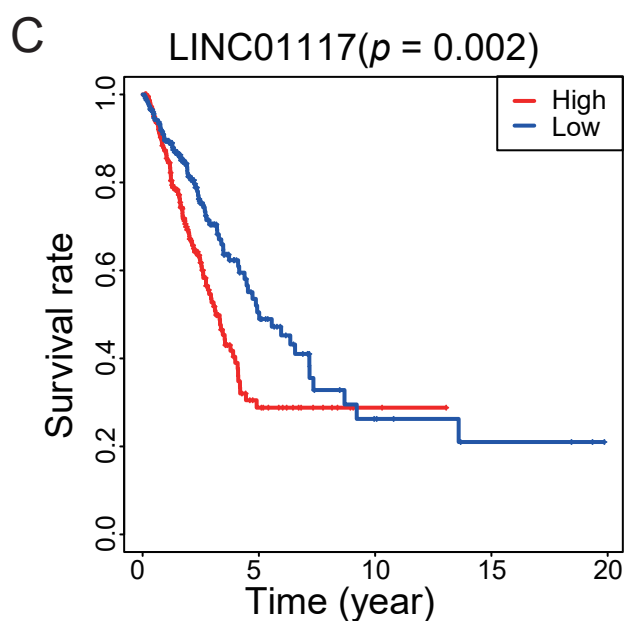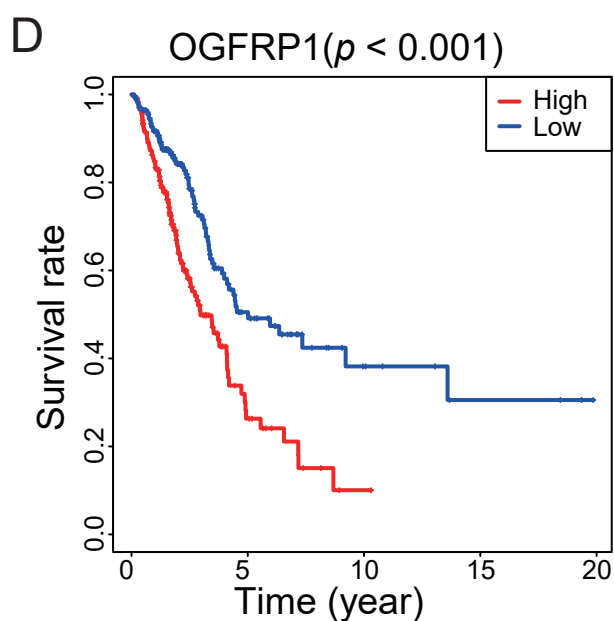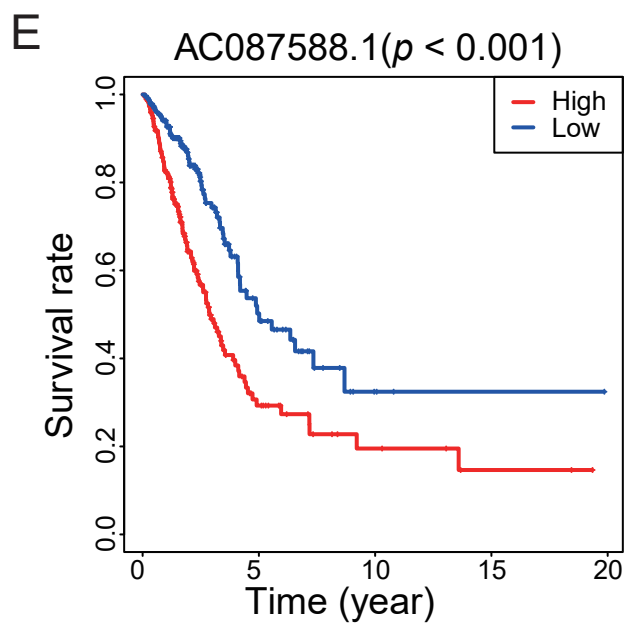

Supplement: Supplementary file 5 [file Image1.pdf]
